# Genetic profile analysis of tumor stem cells in locally advanced breast cancer

**DOI:** 10.1186/1753-6561-7-S2-P69

**Published:** 2013-04-04

**Authors:** Willian A Silveira, Patrícia VB Palma, Renata Sicchieri, Heriton MR Antonio, Larissa RM Mandanaro, Tatiane MG Oliveira, Jurandyr M Andrade, Daniel G Tiezzi

**Affiliations:** 1 Department of Gynecology and Obstetrics, Breast Disease Division, Ribeirão Preto School of Medicine, University of São Paulo, Brazil; 2 National Institute of Science and Technology in Stem Cell and Cell Therapy, Center for Cell Therapy and Regional Blood Center, Ribeirao Preto, Brazil

## Background

Breast carcinoma is a highly prevalent and incident disease. About half the observed cases are diagnosed in later and/or disseminated disease stages. Treatment success in advanced disease stage occurs in about 20% of cases. The identification of patients who would most benefit from neoadjunvant therapy can reduce treatment costs and avoid adverse effects in patients with low probability of response. However, is not yet possible to make such a prediction. The cancer stem cells (CSCs) paradigm relates a cell population resistant to radiotherapy, chemotherapy and cells capable of tumor initiation and recurrence. The identification and characterization of CSCs in the primary tumor can be an effective method of predicting response to neoadjuvant chemotherapy in locally advanced breast cancer.

## Materials and methods

We aim to include 40 patients diagnosed with invasive ductal carcinoma, who will undergo neoadjuvant chemotherapy before surgery. We are in process of collecting tumor tissue biopsies and quantifying, by flow cytometry, and separating, by FACS, the CSCs. We will define CSCs genetic profiles and correlate them with pathological response to the treatment. Biopsies have been collected from five patients to date; from these samples, CSCs were sorted and RNA was extracted and stored. We are following patients for their clinical progress.

## Results

To date, we did not observe statistical differences in the percentage of CSCs in these five samples. We expect to find transcriptional differences between CSCs in tumors from patients who respond to neoadjuvant chemotherapy and from patients who do not respond to treatment regimens.

## Conclusions

Although our preliminary data did not show differences, we expect a slightly different percentage of CSCs in tumor samples from responders *vs*. non-responders, in a larger sample set. However, CSCs transcriptome differences between the two groups of patients may yield a better understanding of neoadjuvant chemotherapy resistance, preventing unnecessary and costly treatment for many patients.

## Financial support

FAPESP.

